# Astrocyte metabolism and signaling pathways in the CNS

**DOI:** 10.3389/fnins.2023.1217451

**Published:** 2023-09-04

**Authors:** Yong-mei Zhang, Ying-bei Qi, Ya-nan Gao, Wen-gang Chen, Ting Zhou, Yi Zang, Jia Li

**Affiliations:** ^1^School of Pharmaceutical Science and Technology, Hangzhou Institute for Advanced Study, University of Chinese Academy of Sciences, Hangzhou, Zhejiang, China; ^2^National Center for Drug Screening, State Key Laboratory of Drug Research, Shanghai Institute of Materia Medica, Chinese Academy of Sciences, Shanghai, China; ^3^University of Chinese Academy of Sciences, Beijing, China; ^4^Institute of Pharmaceutical Sciences, China Pharmaceutical University, Nanjing, Jiangsu, China

**Keywords:** astrocyte, metabolism, neurological disorders, energy imbalance, neural circuit

## Abstract

Astrocytes comprise half of the cells in the central nervous system and play a critical role in maintaining metabolic homeostasis. Metabolic dysfunction in astrocytes has been indicated as the primary cause of neurological diseases, such as depression, Alzheimer’s disease, and epilepsy. Although the metabolic functionalities of astrocytes are well known, their relationship to neurological disorders is poorly understood. The ways in which astrocytes regulate the metabolism of glucose, amino acids, and lipids have all been implicated in neurological diseases. Metabolism in astrocytes has also exhibited a significant influence on neuron functionality and the brain’s neuro-network. In this review, we focused on metabolic processes present in astrocytes, most notably the glucose metabolic pathway, the fatty acid metabolic pathway, and the amino-acid metabolic pathway. For glucose metabolism, we focused on the glycolysis pathway, pentose-phosphate pathway, and oxidative phosphorylation pathway. In fatty acid metabolism, we followed fatty acid oxidation, ketone body metabolism, and sphingolipid metabolism. For amino acid metabolism, we summarized neurotransmitter metabolism and the serine and kynurenine metabolic pathways. This review will provide an overview of functional changes in astrocyte metabolism and provide an overall perspective of current treatment and therapy for neurological disorders.

## 1. Introduction

Astrocytes are remarkably multifunctional cells, and most of their functions are closely connected with neurons in the brain. Astrocytes form a functional syncytial network via their gap junctions and play important homeostatic roles in the central nervous system (Dong et al., 2022). This connection allows for intercellular communication of neurons and astrocytes through various mechanisms, including both chemical and synaptic transmissions (Lines et al., 2020; Shan et al., 2021). Once cast as a supporting role for neurons, recent advances have slowly shifted the views of astrocytes to a more central role. Astrocytes have been found to undergo various changes ranging from hypertrophy, atrophy, or cell death in response to injury and neurological disorders (Verkhratsky et al., 2017b; Escartin et al., 2021). These morphological changes during neurological disorders may alter astrocytic metabolism (Cotto et al., 2019). Recent studies have highlighted the significant impact of astrocyte metabolism on neurological disorders (Muddapu et al., 2020). However, the causal link between astrocytic metabolic dysregulation and the onset of various neurological disorders remains elusive (Phatnani and Maniatis, 2015). In this review, we explore the morphology and functionality of astrocytes, as well as the metabolic alterations they undergo in the context of neurological disorders such as depression, Alzheimer’s disease (AD), and epilepsy. Our goal is to offer innovative perspectives that can guide future research in this field.

## 2. Astrocyte morphology and functionality in the brain

Astrocytes exhibit various morphologies, such as star-shaped, bushy, and spongiform structures, which exist in the brain and spinal cord (Pathak and Sriram, 2023b). However, there are currently controversies over the total number of astrocytes and their proportions in different brain regions. It is estimated that astrocytes make up almost 40% of all cells in the human brain, with variations in different brain regions (Sherwood et al., 2006; von Bartheld et al., 2016). Astrocytes in the CNS are divided into four morphological types: protoplasmic, fibrous, varicose, and interlaminar (Falcone et al., 2019; Rasmussen and Smith, 2022). Protoplasmic astrocytes possess bushy processes and exist primarily in the gray matter. These protoplasmic astrocyte processes extend to the blood vessels, forming a connective membrane that connects to the blood brain barrier (BBB; Jackson et al., 2022). Protoplasmic astrocytes have various functions, including modulation of synaptic function, clearance of glutamate, regulation of blood flowrate, and participation in synaptogenesis (Aten et al., 2022). In contrast, fibrous astrocytes possess long extending processes and are typically distributed in the white matter (Sartoretti and Campetella, 2022). Varicose projection and interlaminar astrocytes are only observed in humans and chimpanzees (Colombo, 2018). Interlaminar astrocytes connect to neurons, pia, and capillaries, suggesting roles such as cortical neuron communication, and may play an essential role in the BBB (Falcone et al., 2019).

The special cytoarchitectural and quantitative features of astrocytes make them play an important role in different metabolic pathways. Structurally, astrocytes are distributed around blood vessels and neurons in the brain, connecting the periphery and the brain for energy exchange and acting as a bridge for communication between cells (Chai et al., 2017; Yue and Hoi, 2023). Regarding glucose metabolism, astrocytes are a primary site for glycolysis and provide neurons with glycogen and lactate, and the astrocyte-neuron lactate shuttle model (ANLS) is critical for neuronal activity (Herrera Moro Chao et al., 2022). Moreover, astrocytes participate in maintaining pathways of amino acid metabolism, fatty acid metabolism, ion and water homeostasis, defense against oxidative stress, and anti-inflammation (Sofroniew, 2020). Changes in these astrocytic pathways also influence the activity of neurons and may lead to neurological disorders (Dzyubenko and Hermann, 2023; Patani et al., 2023; Yao et al., 2023).

## 3. Glucose metabolism

### 3.1. Astrocytes and the glucose metabolism pathway: main energy source of the brain

**Figure 1:**
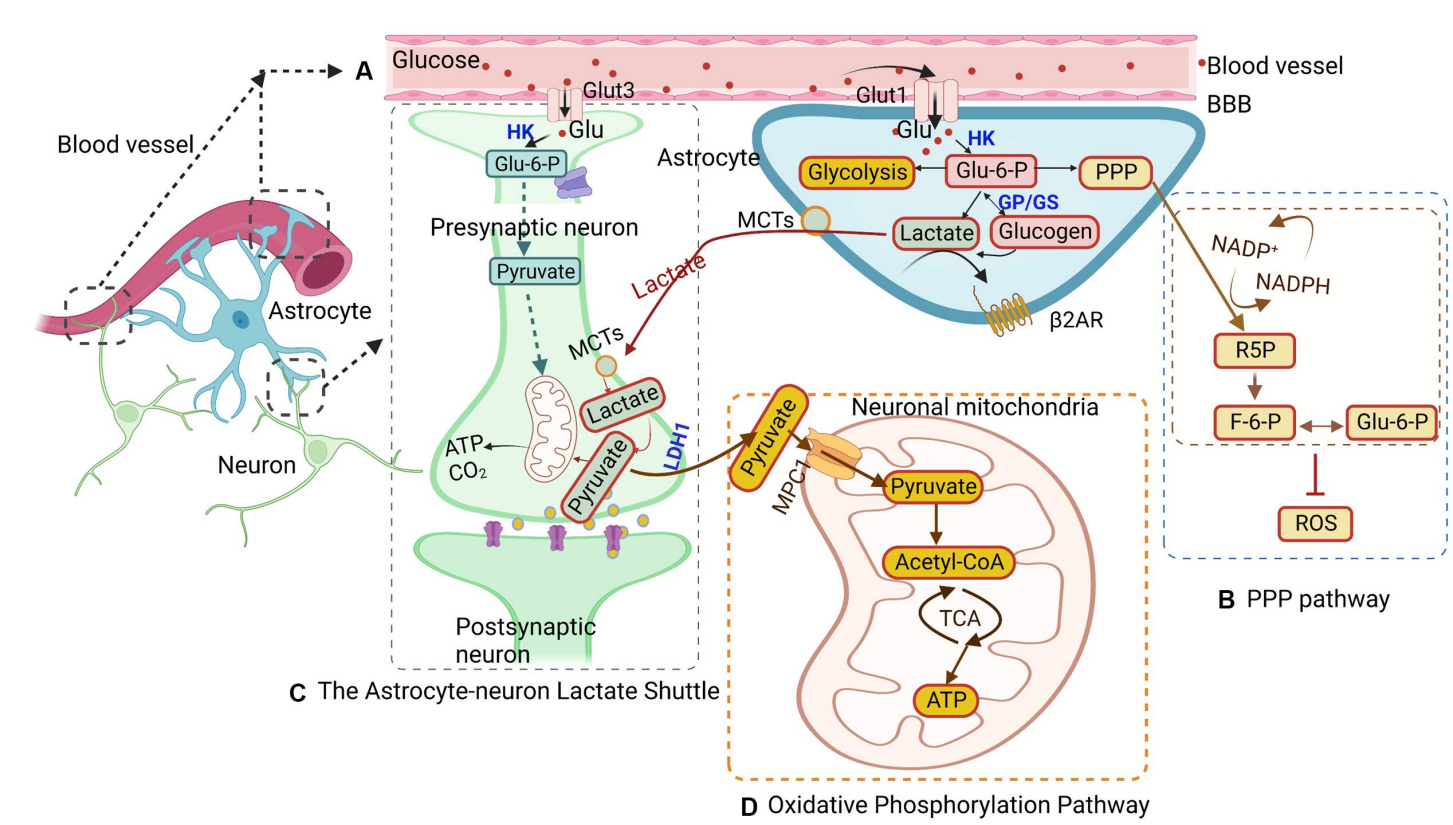
Glucose metabolism in astrocytes. **(A)** Glucose is transported from the blood brain barrier (BBB) to astrocytes and neurons through glucose transporters (GLUTs) and phosphorylated to glucose-6-phosphate (G6P). G6P enters different pathways, such as glycolysis, the astrocyte-neuron lactate shuttle, the pentose-phosphate pathway (PPP), and the oxidative phosphorylation pathway. **(B)** In the PPP, G6P catalyzes ribulose-5-phosphate (R5P), converting nicotinamide adenine dinucleotide phosphate (NADP) + to NADPH at the same time. Concurrently, R5P can also be converted to glyceraldehyde-3-phosphate and fructose-6-phosphate (F6P), the latter of which can isomerize back to G6P. **(C)** The astrocyte-neuron lactate shuttles provide energy for neuron activity. Lactate is transferred from astrocytes into neurons through monocarboxylic acid transporters (MCTs) and is converted to pyruvate to generate ATP in mitochondria. **(D)** The oxidative phosphorylation pathway in astrocytes converts G6P to pyruvate, which undergoes oxidative decarboxylation to form acetyl-CoA and then enters the tricarboxylic acid (TCA) cycle to generate ATP in mitochondria. MPC1, mitochondrial pyruvate carrier 1; HK, hexokinase; GP, glycogen phosphorylase; LDH1, lactate dehydrogenase 1.

Astrocytes metabolize glucose from the bloodstream to fuel surrounding neurons (Figure 1). Glucose is regulated mainly by glucose transporters (GLUT; Mergenthaler et al., 2013). These transporters, such as GLUT1 and GLUT3, are abundant in astrocytes and neurons, respectively, while astrocytes show limited GLUT2 expression (Koepsell, 2020; Figure 1A). There are currently two types of GLUT1 isoforms. The first GLUT1 isoform is the 55-kDa isoform, which is located in the endothelial cells that form the BBB (Kreft et al., 2012). Glucose enters astrocytes from the interstitium via the 45-kDa isoform of GLUT1 and into neurons via GLUT3, a neuronal GLUT (Kreft et al., 2012). GLUT 1 transporters are located in the astrocyte cell body and foot processes, which shuttle glucose from the bloodstream across the BBB into astrocytes (Nguyen et al., 2021). GLUT3 is located in the neural foot processes and transports glucose into neurons. Following its entry into the cell, glucose undergoes phosphorylation, a process catalyzed by hexokinase type I, which is ubiquitous in the brain and closely associated with mitochondria (Koepsell, 2020). Hexokinase type I migrates from mitochondria to microtubules during gap junction inhibition, inducing the expression of hexokinase type II and GLUT3, which are normally not present in astrocytes (Sánchez-Alvarez et al., 2004). Postphosphorylation, glucose becomes glucose-6-phosphate (G6P), which then enters either glycolysis or the pentose-phosphate pathway (PPP; Takahashi, 2021). During glycolysis, G6P is converted into fructose-6-phosphate (F6P) by phosphohexose isomerase and subsequently phosphorylated by phosphofructokinase to yield fructose 1,6-bisphosphate (F1,6-bisP). Aldolase then cleaves F1,6-bisP to generate glyceraldehyde 3-phosphate (Gly3-P) and dihydroxyacetone phosphate (DHAP), which can be interconverted by phosphotriose isomerase. Gly3-P undergoes conversion into 1,3-bisphosphoglycerate (1,3-bisPG) through a process catalyzed by nicotinamide adenine dinucleotide (NAD)-dependent dehydrogenase and is then phosphorylated by phosphoglycerate kinase into 3-phosphoglycerate (3-PG). 3-PG is dephosphorylated to form 2-phosphogylcerate by phosphoglycerate mutase and subsequently dehydrated by enolase into phosphoenolpyruvate. Phosphoenolpyruvate is phosphorylated by pyruvate kinase into pyruvate, which can then enter the Krebs cycle or be converted to lactate. Both of these routes can generate NADH that can be used for continuous glycolysis, the former being more complicated due to transport out of the mitochondria (Kumari, 2018). Alternatively, pyruvate can be converted into acetyl-coenzyme A (acetyl-CoA) by the pyruvate dehydrogenase complex, serving as a precursor for the synthesis of amino acids, phospholipids, ketone bodies, and other substrates (Shi and Tu, 2015). Glucose metabolism in astrocytes provides the necessary metabolic substrate to respond to the energy needs of neurons, ensuring their normal functions.

Another pathway for G6P is the PPP pathway, which accounts for approximately 3% of glucose metabolism (Figure 1B; Takahashi, 2021). The PPP is a shunt pathway split into two phases: oxidative and nonoxidative (Kamada et al., 2003). The oxidative phase generates ribulose-5-phosphate (R5P) through G6P dehydrogenase and assists in neutralizing radical oxygen species (ROS; Wamelink et al., 2008; Takahashi, 2021). Meanwhile, in the nonoxidative phase, R5P can be isomerized into ribose-5-phosphate and used for nucleotide biosynthesis (Wamelink et al., 2008). Additionally, R5P can be converted into glyceraldehyde-3-phosphate and fructose-6-phosphate (F6P), with the latter being able to isomerize back to G6P (Wamelink et al., 2008). The PPP has been identified as a mechanism for protecting neurons from oxidative stress (Bolanos and Almeida, 2010). Interestingly, the rate of glucose entry into the PPP in astrocytes is five to seven times higher than that in neurons, reflecting astrocytes’ higher glycolytic rate (Takahashi, 2021). Under hypoxic conditions, the rate of glucose flux into the PPP in astrocytes is elevated, while PPP activity is decreased in cultured neurons (Takahashi, 2021). These observations underscore the critical role that astrocytes play under hypoxic conditions, providing antioxidant defense for neurons to help prevent neuronal cell death.

In situations of glucose availability, G6P is shunted into glycogen storage via conversion into glycogen by glycogen-synthase for later use (Wender et al., 2000). When astrocytes or neurons require energy, glycogen phosphorylase can revert glycogen back into G6P, allowing it to reenter glycolysis (Nadeau et al., 2018). Notably, astrocytic glycogen is not uniformly distributed, and research suggests that it tends to accumulate in areas of the brain with the highest synaptic density (Phelps, 1972). This finding indicates that glycogen may play a role in synaptic functionality. However, subsequent research has revealed significant glycogen concentrations in the white matter as well, which does not contain synapses (Cruz and Dienel, 2002). Given that the white matter region consists of glial cells and is devoid of neurons, glycogen stored in this region may serve a supportive role for myelin or function as storage. Early research primarily considered glycogen as a safeguard against hypoglycemia, providing the brain with energy during periods of low glucose or when the glucose present in the blood is insufficient to meet increased energy demand (Brown et al., 2003).

Recent research has unveiled the versatile role of glycogen in the brain’s energy dynamics. It has been discovered that glycogen can be converted to lactate, power glutamate transport, and contribute to the synthesis of glutamine, a precursor to glutamate, a key neurotransmitter essential for neuronal communication (Brown et al., 2004; Gibbs et al., 2006). These findings imply that glycogen may have a significant and multifaceted role in neuronal modulation through the process of glycogenolysis. Moreover, this implies that glycogen can be converted into other energy substrates, such as glucose and lactate, whenever necessary for maintaining brain functionality.

### 3.2. Astrocytes and lactate: functionality in neuronal regulation

In astrocytes, lactate is generated as a byproduct of glycolysis. Lactate is a critical energy substrate produced by astrocytes during neuronal activity (Figure 1C; Roberts and Chih, 2003; Xue et al., 2022). One hypothesis suggests that the synaptic release of glutamate can trigger glycolytic production of lactate in astrocytes. The lactate produced is then released extracellularly and taken up by surrounding neurons to fuel oxidative metabolism during activity (Pellerin and Magistretti, 1994). This hypothesis was indirectly supported by the distribution of lactate dehydrogenase isoforms in activity-dependent astrocytes (Pellerin et al., 1998). However, PET measurements of cerebral oxygen consumption in the brain suggest that neurons increase their oxidative metabolism in parallel with an increase in pyruvate (Kasischke, 2009). This implies that glycolysis in neurons, not astrocytes, determines the kinetics of the metabolic response. More recent research found that lactate can act as a viable energy source and increase in the brain during neuronal activity, suggesting that it may replace glucose as the primary energy source for neurons (Wyss et al., 2011). Additionally, lactate produced from glucose or glycogen in astrocytes can be transferred via monocarboxylic acid transporters (MCTs) from astrocytes to neurons or so-called ANLS (Magistretti and Allaman, 2018; Yamagata, 2022). It is theorized that these shuttles shift between astrocytes, providing neurons with the necessary energy for normal operations. However, whether neurons prefer lactate over glucose remains undetermined. Lactate is released by astrocytes through MCTs into the extracellular matrix, from which it may be transported into neurons via MCTs present on neurons or passively through gap junctions (Dienel, 2019; Yamagata, 2022). It is theorized that astrocytes only release lactate to neurons during periods of low energy or as a supplementary energy source during neuronal activity, as suggested by research studies (Barros, 2013; Magistretti and Allaman, 2018). Once lactate enters the neurons, it is converted back into pyruvate and transported into the mitochondria to generate ATP (Alberini et al., 2018).

Although there have been theories on whether neurons require lactate as an energy source, Mangia et al. showed that neurons export lactate and astrocytes import lactate and for enabling astrocytes to export lactate, the glucose transport capacity of astrocytes must be increased 12-fold and that glucose must not respond to activation with increased glycolysis (Mangia et al., 2009). Furthermore, in a more recent study, Diaz-Carcia et al. measured the neuronal NADH/NAD^+^ ratio by employing a biosensor during stimulation and found that neurons upregulate glycolysis more than oxidation and release lactate (Diaz-Garcia et al., 2017). These findings indicate that activated neurons do not depend on extracellular lactate for neuronal function, which questions the theory of ANLS at the cellular level (Dienel, 2019). Although extracellular lactate is not used for energy supplementation for neuronal firing, there may be other functionalities of lactate in the brain. For instance, astrocytes have recently been found to contribute to memory formation (Kol et al., 2020) and employ lactate in influencing memory or cognitive behaviors. Recent studies have revealed that lactate production in astrocytes expresses β2 adrenergic receptors (β2AR), which are integral for memory consolidation (Alberini et al., 2018). Furthermore, disruption of the astrocyte-neuron lactate shuttle was found to impair the formation of long-term memory (Lindberg et al., 2019). These findings underscore the importance of lactate production by astrocytes and its influence on cognitive functions. Lactate was also found to signal through specific G-protein coupled receptors expressed in neurons and glial cells, suggesting a possible role in neurotransmission, neurovascular coupling, and brain energy metabolism (Morland et al., 2015).

### 3.3. Oxidative phosphorylation pathway: mitochondrial metabolism in astrocytes

The oxidative phosphorylation pathway is present in both astrocytes and neurons. Although this pathway is more prominent in neurons, astrocytes utilize oxidative phosphorylation to protect neurons against oxidative stress by providing neurons with a reduced form of glutathione (Takahashi, 2021; Figure 1D). This pathway takes place in the cell’s mitochondria and is vital for maintaining cellular functionality. The mitochondrion, a small organelle in the cell responsible for energy generation, is found in the processes of astrocytes (Jackson and Robinson, 2018). The pyruvate generated from glycolysis is actively transported into the mitochondria via mitochondrial pyruvate carrier 1 (Rose et al., 2020). Pyruvate undergoes oxidative decarboxylation, forming acetyl-CoA, which then enters the tricarboxylic acid (TCA) cycle. Upon reacting with oxaloacetate, citrate is formed, and a series of oxidation reactions generate ATP (Rose et al., 2020). Although the oxidative phosphorylation pathway can produce energy in times of stress to aid in the survival of astrocytes and neurons, other pathways have also been found to be capable of sustaining astrocyte survival in the event of mitochondrial inhibition (San Martin et al., 2017). This activates 5’-AMP-activated protein kinase (AMPK) to upregulate the glycolysis of 6-phosphofructo-1-kinase (PFK1), which compensates for the loss of mitochondrial ATP and maintains the mitochondrial membrane potential (Almeida et al., 2004). During times of low energy production, such as under ischemic conditions, metabolic shifts occur from neurons to astrocytes to preserve energy due to the lower energy demand of astrocytes compared to neurons (Deitmer et al., 2019). Liang et al. found that GLUT3 presents unique Michaelis–Menten characteristics of low K_m_ and high V_max_, indicating that GLUT3 can uptake glucose from the extracellular fluid of low glucose concentration by the highest possible maximum velocity (Liang and Bourdon, 2018). Therefore, GLUT3 on neurons is beneficial for glucose uptake at low glucose concentrations in the brain. In this condition, the glucose concentration of the brain parenchyma was maintained at 1–2 mM. Inhibition of the oxidative phosphorylation pathway in neurons can lead to cell death because glycolysis in neurons cannot be activated to the same extent as in astrocytes (Bolanos et al., 1995). This activation of glycolysis is partially due to the presence of 6-phosphofructose-2-kinase/fructose-2,6-bisphosphatase-3 (PFKFB3), a key enzyme promoting glycolysis (Almeida et al., 2004). Nonetheless, the oxidative phosphorylation pathway is essential in providing energy for both astrocytes and neurons, ensuring neuronal survival and maintaining functionality (Bonvento and Bolanos, 2021).

## 4. Fat and lipid metabolism

### 4.1. Astrocytes and fatty acid metabolism

**Figure 2:**
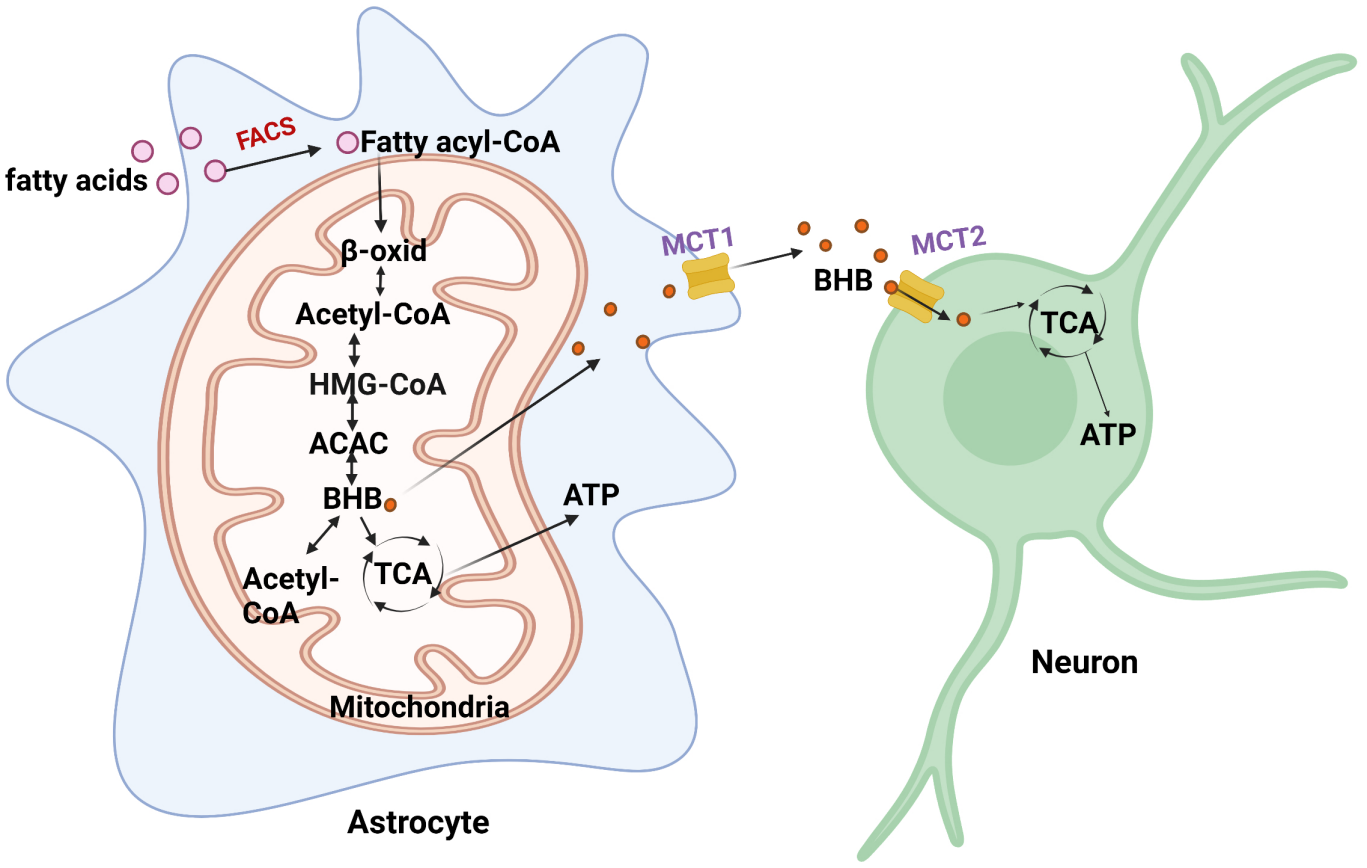
Fatty acid metabolism in astrocytes. When energy is scarce, fatty acids are converted to fatty acyl-CoA and undergo β-oxidation (β-oxid) to produce β-hydroxybutyrate (BHB). BHB converts back to acetyl-CoA via β-ketoacyl-CoA transferase or enters the TCA cycle to generate ATP. It then enters the TCA cycle to generate ATP. FACS, fatty acyl-CoA synthetase; HMG-CoA, 3-hydroxy-3-methylglutaryl coenzyme A; ACAC, acetoacetate.

Astrocytes are the main sites for fatty acid oxidation in the brain (Edmond et al., 1998). During energy deficits, fatty acid oxidation and ketone body production are essential in the brain as an alternate source of energy for maintaining normal brain functions (Figure 2; Le Foll and Levin, 2016). Fatty acids can help support the TCA cycle and oxidative phosphorylation in astrocytes (Panov et al., 2014). In the TCA cycle, α-ketoglutarate is converted into succinyl CoA through ketoglutarate dehydrogenase, after which the coenzyme is removed through succinyl CoA synthetase to form succinate. The formation of succinate and CoA allows for the phosphorylation of GDP to GTP.

### 4.2. Astrocytes and ketogenic metabolism

Astrocytes have the capacity to take up, synthesize, and release β-hydroxybutyrate (BHB; Le Foll and Levin, 2016). Astrocytes are the only source of ketone body (KB) production in the brain (Figure 2; Le Foll and Levin, 2016). One way for the brain to obtain KBs is from the BBB through monocarboxylate transporter 1 (MCT1) in endothelial cells, oligodendrocytes, and astrocytes. The liver supplies most of the KBs in the BBB and are oxidized by the brain when circulating glucose becomes scarce. Particular conditions, including prolonged fasting, uncontrolled diabetes, and breastfed newborn babies, increase circulating BHB and acetoacetate (Jensen et al., 2020). In such cases, the brain slowly adapts to the use of KBs to preserve neuronal synaptic function and structural stability. In the ketogenic synthetic pathway of astrocytes, fatty acids are transported into the mitochondria and then converted into acetyl-CoA through the β-oxidation cycle. Two acetyl-CoAs are then converted into acetoacetyl-CoA through acetyl coenzyme A acetyltransferase (ACAT). Acetoacetyl-CoA is then converted to 3-hydroxy-3-methylglutaryl coenzyme A (HMG-CoA), followed by acetoacetate, and finally, β-hydroxybutyrate (BHB) is supplied as a substrate for neuronal ATP synthesis. Astrocytes and, more recently, oligodendrocytes have been found to express MCT1 (Lee et al., 2012). MCT1 expression was found to be higher in oligodendrocytes than in astrocytes (Lee et al., 2012). Neurons almost exclusively express the MCT2 isoform, which possesses a high affinity for BHB released from endothelial cells and astrocytes. Acetoacetate and BHB are two ketone bodies used for energy when glucose levels decrease in neurons. Upon entering neurons, BHB can be converted to acetoacetate via β-hydroxybutyrate dehydrogenase, which is then converted back to acetyl-CoA via β-ketoacyl-CoA transferase, which subsequently enters the TCA cycle (Dhillon and Gupta, 2022). An interesting study investigating substrate oxidative metabolism in brain cellular models showed that oxidation of KBs by neurons and oligodendrocytes is three times more efficient than that by astrocytes (Edmond et al., 1987).

In addition to serving as an energy supply, KBs also serve as substrates for the production of lipids in the brain, such as myelin (Cunnane and Crawford, 2014). A study reported that ketone bodies protect myelin-forming oligodendrocytes and reduce axonal damage (Mu et al., 2022). Moreover, KBs can also act as posttranslational modification proteins to activate intracellular signaling pathways (Koppel and Swerdlow, 2018). Research has found that MCT2 expressed in neurons is mainly colocalized to mitochondria-rich postsynaptic density structures, suggesting that KBs play an important role in synaptic transmission (Pierre et al., 2002). Neurotransmitters released by neurons during enhanced synaptic activity may interact with astrocytes, stimulating the production of lactate and ketones for cellular activity (Guzman and Blazquez, 2001). Studies have shown that glutamate can enhance ketogenesis in cultured astrocytes, a process dependent on glutamate transporters (Guzman and Blazquez, 2004). Further research has found that ketones can modulate neuronal firing by opening ATP-sensitive calcium channels (Ma et al., 2007). This indicates the significant role of ketones in regulating neuronal activity, which might explain why a ketogenic diet is an effective treatment for epilepsy and other neurological disorders.

### 4.3. Sphingolipid metabolism: astrocytic regulation of neuron metabolism

Sphingolipid metabolism, although occupying a relatively small part of metabolism, plays an essential role in the brain. These sphingolipids are critical components in the formation of myelin sheaths. The biosynthesis of sphingolipids entails the conversion of L-serine and palmitoyl-CoA into ceramide, which is a crucial substrate for the generation of other sphingolipids, such as ceramide-1-phosphate (C1P) and sphingosine. Sphingosine can then be further converted into sphingosine-1-phosphate (S1P; Pralhada Rao et al., 2013). Both ceramide and sphingosine are vital regulators of stress responses, possessing the capability to inhibit cellular proliferation and mediate apoptosis, growth arrest, senescence, and differentiation (Pralhada Rao et al., 2013). On the other hand, S1P presents contrasting functionality to its unphosphorylated counterpart by promoting cell proliferation, migration, angiogenesis, and cell survival (Zeidan and Hannun, 2007). Early research has identified the critical role of sphingolipids in brain development and neuron survival (Hirabayashi and Furuya, 2008). Moreover, sphingolipid metabolism might also be instrumental in regulating astrocytic metabolic support for neurons (Lee et al., 2022). Given that these glycoproteins possess numerous essential functions for maintaining astrocyte and neuron stability within the brain, they represent an important factor to consider in the study and treatment of neurological disorders.

## 5. Amino acid metabolism in astrocytes

### 5.1. The glutamate/GABA-glutamine cycle

Similar to the energy metabolic pathways, the amino acid metabolic pathway in astrocytes plays an instrumental role in modulating brain functionality. Astrocyte metabolism is closely connected with the glutamate/GABA-glutamine cycle in neurons and helps regulate neurotransmitter homeostasis (Augusto-Oliveira et al., 2020). Astrocytes can take up synaptically released neurotransmitters, such as glutamate and γ-aminobutyric acid (GABA), and metabolize them into glutamine, which returns to neurons (Albrecht et al., 2007). Glutamate is essential for synaptic functionality within the brain and is also identified as a precursor for GABA (Roberts and Frankel, 1950). The exchange of glutamate, GABA, and glutamine between neurons and astrocytes is known as the glutamate/GABA-glutamine cycle, which is crucial for maintaining excitatory and inhibitory neurotransmission (Andersen et al., 2022).

**Figure 3:**
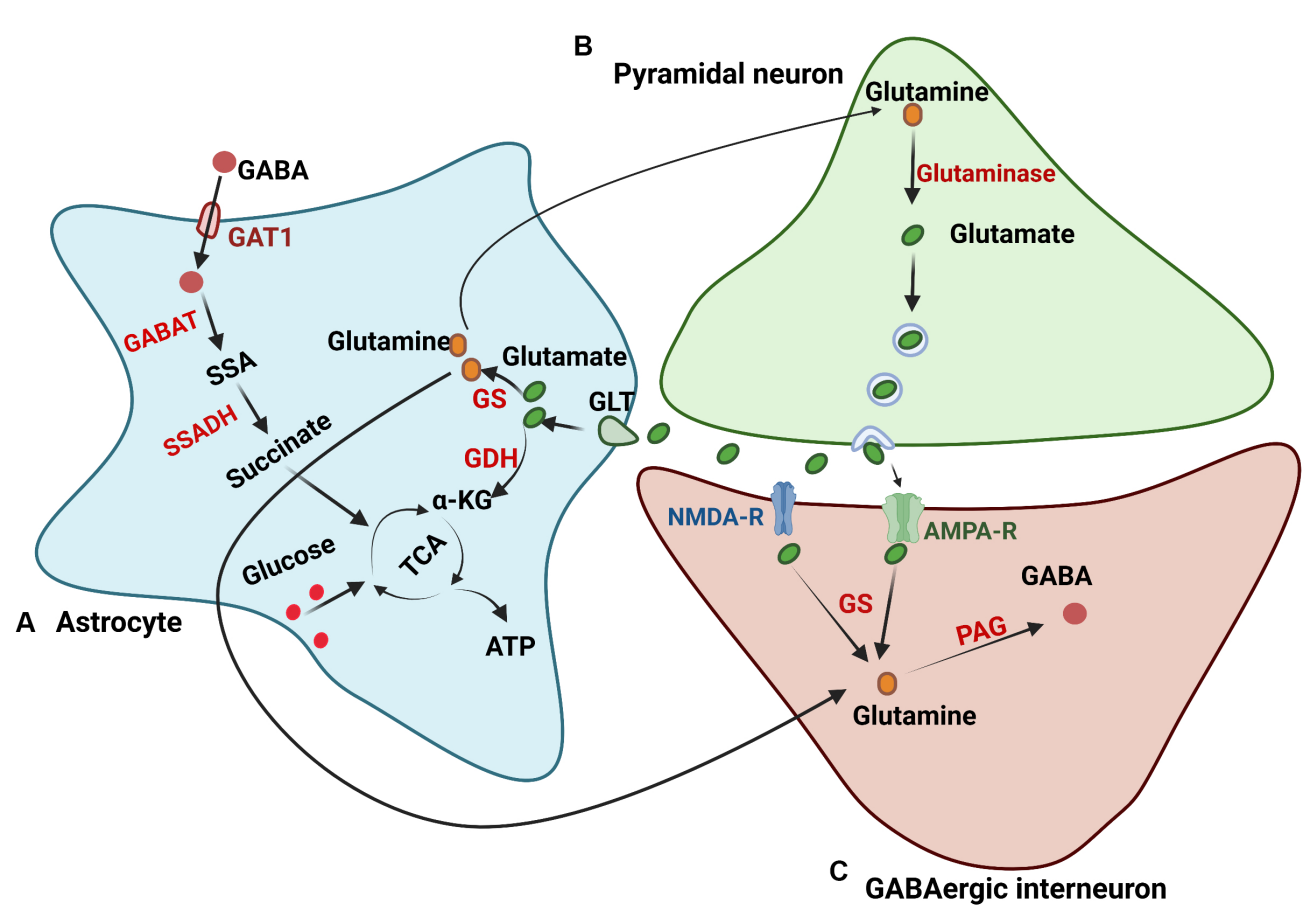
Glutamate and GABA metabolism. **(A)** Astrocytes regulate glutamate in the brain through glutamate transporters (GLTs). After being transported in astrocytes, glutamate undergoes the glutamine synthase pathway to produce glutamine or the TCA cycle to generate ATP. **(B)** The glutamate synthesis pathway in neurons. Glutamine in neurons is transported from astrocytes and is deaminized to generate glutamate by glutaminase. **(C)** GABA synthesis in neurons. Glutamine in neurons for GABA synthesis is transported from astrocytes or synthesized from glutamate, which is released from the excitatory synapse. Glutamine is then converted to GABA through phosphate-activated glutaminase (PAG). GS, glutamine synthetase; GDH, glutamate dehydrogenase; GABAT, GABA transaminase; GAT1, GABA transporter 1; SSA, succinyl semialdehyde; SSADH, semialdehyde dehydrogenase.

Importantly, efficient synaptic glutamate uptake, which is mainly transported by glutamate transporters of brain excitatory amino acid transporter 1 (EAAT1) and EAAT2 in astrocytes, is vital to avoid excitatory overstimulation and concurrent excitotoxic damage (Storck et al., 1992; Petr et al., 2015). Reports have found that astrocytes have a greater ability than neurons to take up glutamate, potentially because astrocytes can maintain a more stable membrane potential with high extracellular Na^+^ and low K^+^ compared to neurons, and neuronal firing makes neurons have a less stable Na^+^/K^+^ ratio (Mahmoud et al., 2019). Some electrophysiological studies have shown that the inward transport of 3 Na^+^ and 1 H^+^ ions with each glutamate anion drives the outward transport of 2 K^+^ ions, relying on their concentration gradients (Levy et al., 1998). Moreover, the transport of many other ions, such as Cl^−^ and H^+^, may not directly drive glutamate uptake but may cause changes in the ionic concentrations within astrocytes (Untiet et al., 2017). Upon transportation into astrocytes, glutamate either follows the glutamine synthase pathway, wherein it converts into glutamine, or enters the TCA cycle where it converts to α-ketoglutarate, a substrate for ATP production (Waniewski and Martin, 1986). The preference between these two pathways is contingent on the extracellular concentration of glutamate (Anderson and Swanson, 2000). If the concentration is less than 0.2 mM, glutamate is metabolized into glutamine for reuse, while oxidative metabolism is favored if the glutamate concentration surpasses 0.2 mM (McKenna et al., 1996). Glutamine, which is released into the extracellular space by astrocytes, is imported into glutamatergic and GABAergic neurons to synthesize glutamate and GABA, respectively. When acting as a precursor for GABA synthesis, glutamine is converted to GABA via phosphate-activated glutaminase (PAG; Schousboe et al., 2013). In addition, some studies have shown that astrocytic release of glutamate to the surrounding neurons helps to synchronize their firing and modulate their excitatory transmission (Harada et al., 2015). Subsequent studies found that the elevation of intracellular Ca^2+^ in astrocytes induced glutamate release from astrocytes (Fellin et al., 2004), further expanding our understanding of astrocyte functionality (Figure 3).

Astrocytes are also involved in the uptake and metabolism of GABA synaptically through high-affinity GABA transporters (GATs; Scimemi, 2014), and GAT3 is mainly expressed in astrocytes among the GATs (Melone et al., 2015). Through coupling to the cotransport of 1 Cl^−^ and 3 Na^+^, GABA can be transported into astrocytes but shows no stimulation of astrocyte metabolism (Chatton et al., 2003). GABA is then oxidized in astrocytes through the transfer of nitrogen to other amino acids via GABA transaminase (GABA-T), forming succinic semialdehyde. Subsequently, succinic semialdehyde is converted into succinate via succinic semialdehyde dehydrogenase (SSADH) and enters the TCA cycle (Andersen et al., 2020). Brain GABA metabolism is essential, and reports suggest that malfunctions of GABA-T and SSADH can cause severe encephalopathies (Malaspina et al., 2016; Koenig et al., 2017), and GABA metabolism in astrocytes plays an important role in supporting the synthesis of glutamine (Andersen and Jakobsen, 2020). In addition to being metabolized in astrocytes, GABA can also be synthesized and released from astrocytes. Studies on cultured astrocytes showed that astrocytes can synthesize GABA using glutamate decarboxylase (GAD67) or polyamine putrescine, and the results were also verified *in vivo* (Woo et al., 2018; Kwak et al., 2020). All studies have demonstrated that GABA concentrations are strongly modulated by astrocytes to maintain neurotransmitter balance (Kilb and Kirischuk, 2022).

Glutamine synthesis is very important for astrocyte energy metabolism, and the astrocytic glutamine supply is crucial for neuronal function in the brain. The inhibition of glutamine synthesis can lead to disturbances in both excitatory and inhibitory transmission (Ortinski et al., 2010; Tani et al., 2014). α-ketoglutarate in the TCA cycle is the precursor of glutamine, and impaired TCA cycle function and astrocyte glutamine transfer influence the supply of glutamine for glutamate and GABA synthesis, leading to functional disruption in the brain (Zhou Y. et al., 2019; Cheung and Bataveljic, 2022). This intricate interplay between glutamate, glutamine, and GABA underscores the critical role of astrocytes in modulating neuronal functionality (Figure 3).

### 5.2. Glutathione: an important intermediary in the maintenance of the intracellular redox balance

Glutathione (GSH) is a tripeptide that serves as a critical antioxidant in the brain and affects multiple cellular functions (Iskusnykh et al., 2022), especially in astrocytes (Pérez-Sala and Pajares, 2023). GSH consists of cysteine, glutamic acid, and glycine residues and is widely distributed throughout the CNS. The synthesis of GSH is consistent across different tissues (Dringen et al., 2015). Initially, glutamic acid and cysteine serve as substrates to generate glutamylcysteine (γGluCys) by γ-glutamylcysteine synthetase. GSH is then produced from glycine and γGluCys by glutathione synthetase (Segura-Aguilar et al., 2022). The GSH system contains exogenous GSH, GSH synthesis, and GSH recycling (Pérez-Sala and Pajares, 2023). The maintenance of the GSH system is critical for the regulation and utilization of reactive oxygen and nitrogen species (Pérez-Sala and Pajares, 2023). GSH may affect many important signaling pathways in the CNS, including neurotransmission, enzyme activation, metal transport in cells, cellular differentiation and proliferation, and apoptosis (Aoyama and Nakaki, 2013). Impaired GSH synthesis leads to disrupted cell signaling and an increased risk of neurological diseases (Aoyama and Nakaki, 2013).

### 5.3. Serine metabolism: cross-communicating metabolism between astrocytes and neurons

L-serine and D-serine, the amino acids akin to glutamine, play a fundamental role in neuron–glia communication (Wolosker, 2011). These two amino acids are essential for excitatory neurotransmission within the central nervous system (CNS; Hashimoto et al., 1992; Wolosker et al., 2008). L-serine is biosynthesized from the glycolytic intermediate 3-phosphoglycerate (Yamasaki et al., 2001). 3-Phosphoglycerate is oxidized by phosphoglycerate dehydrogenase (Phgdh) using NAD^+^ to form 3-phosphohydroxypyruvate, which is then converted to phosphoserine in a transamination reaction catalyzed by 3-phosphohydroxypyruvate aminotransferase (Psat). Phosphoserine is finally dephosphorylated by 3-phosphoserine phosphatase (Psph), generating L-serine. Both *in vitro* and *in vivo* experiments suggest that Phgdh mRNA is mainly expressed in astrocytes and minimally expressed in neurons (Furuya et al., 2000). These findings also strongly suggest that L-serine in the CNS is exclusively synthesized by astrocytes. After being synthesized from glucose in astrocytes, L-serine is shuttled to neurons to fuel the synthesis of D-serine, and the serine shuttle mechanism adds to other possible forms of metabolic interchange between astrocytes and neurons (Wolosker and Radzishevsky, 2013).

D-serine is synthesized from L-serine, and a constant supply of L-serine is critical for D-serine synthesis (Wolosker et al., 2017). L-serine is supplied by astrocytes and transported into neurons through the serine shuttle mechanism (Wolosker et al., 2016). Astrocytic L-serine is shuttled to neurons and is crucial for sustaining neuronal synthesis of D-serine. Once L-serine is inside the neurons, mainly in glutamatergic neurons, it is converted into D-serine through the action of the serine racemase (SR) enzyme (Neame et al., 2019). This D-serine is then released during membrane depolarization. Additionally, D-serine released by neurons can also be absorbed by astrocytes for storage and subsequent activity-dependent release. Notably, D-serine plays a crucial role in pyruvate generation (Wolosker, 2011). Additionally, a study found that neuronal release of D-serine modulates N-methyl-D-aspartate receptor (NMDAR) function, and some of the D-serine produced by neurons might be transported into astrocytes and metabolized via the peroxisomal D-amino acid oxidase (DAO) enzyme (Wolosker and Radzishevsky, 2013). Many studies have demonstrated the importance of endogenous D-serine in mediating NMDAR activation for contextual and working memory in rodents (Balu et al., 2016; Kaplan et al., 2018). The serine shuttle mechanism provides an important relationship between astrocytes and NMDAR function. Taken together, the serine pathway underscores the important role of astrocytes in neuronal functionality.

### 5.4. Kynurenine metabolism: the link between kynurenine metabolism and astrocytes

The kynurenine pathway (KP), responsible for the breakdown of tryptophan into kynurenine and its subsequent conversion into quinolinic acid, picolinic acid, acetyl-CoA, and NAD, plays a critical role in the production of cellular energy through NAD formation. The KP occurs in astrocytes, neurons, macrophages, glia, and so on (Savitz, 2020). The pathway in which tryptophan degrades into kynurenine is known as the kynurenine pathway (KP) and is one of the major regulatory mechanisms of the immune response (Lim et al., 2017). Some inflammatory mediators, such as IFN-γ, TNF-α, lipopolysaccharide (LPS), and viral proteins, can activate indoleamine 2,3 dioxygenase (IDO-1), subsequently activating the KP. The diverse products of kynurenine contribute to a range of functions related to neuron protection. Among kynurenic acids, L-kynurenine (L-KYN) is produced and plays a key role in the neurotoxic and neuroprotective directions of the pathway (Joisten et al., 2021). 4-Hydroxyquinoline-2-carboxylic acid (KYNA), which is a neuroprotective kynurenic acid, is formed directly from L-KYN in astrocytes. The production of KNYA is directly related to increased activity of kynurenine aminotransferases (KATs; Dezsi et al., 2015). A study found that KAT1/2 is mainly expressed in human astrocytes, converting KYN to KYNA, suggesting that astrocytes are the primary site for KYNA production in the brain.

Kynurenine conversion to kynurenic acid, for instance, can have neuroprotective effects by inhibiting ionotropic glutamate receptors at high concentrations and mitigating the activity of glycine on the NMDA receptor (Kessler et al., 1989). Research has shown that even at low concentrations, kynurenic acid can significantly impact glutamate levels (Carpenedo et al., 2001). Moreover, kynurenic acid can modulate cyclic adenosine monophosphate (cAMP) production by enhancing orphan G-protein-coupled receptor activity, thus suppressing several inflammatory pathways (Wirthgen et al., 2017). However, excessive concentrations of kynurenic acid may induce NMDA receptor hypofunction in cortical GABA interneurons, causing disinhibition of glutamate projections (Savitz, 2020). In contrast, quinolinic acid can induce cytotoxicity in neurons by hindering astrocyte glutamate reuptake (Stone and Perkins, 1981). Additionally, it can generate reactive oxygen species, disrupt the BBB, destabilize the cell cytoskeleton, promote tau phosphorylation, and disrupt autophagy (Savitz, 2020). Astrocytes, as noted in earlier reports, express most of the enzymes in the kynurenine pathway, except kynurenine-OHase, and can both produce and degrade quinolinic acid (Guillemin et al., 1999). Moreover, astrocytes can trigger kynurenine pathway activation, leading to the production of L-kynurenine, which can then be used to produce kynurenic acid (Guillemin et al., 1999). These observations underscore the critical role of astrocytes in supporting neuronal survival. By managing these metabolic pathways, astrocytes may help prevent the onset of neurological disorders.

## 6. Astrocyte metabolic pathways in neurological disorders

**Figure 4:**
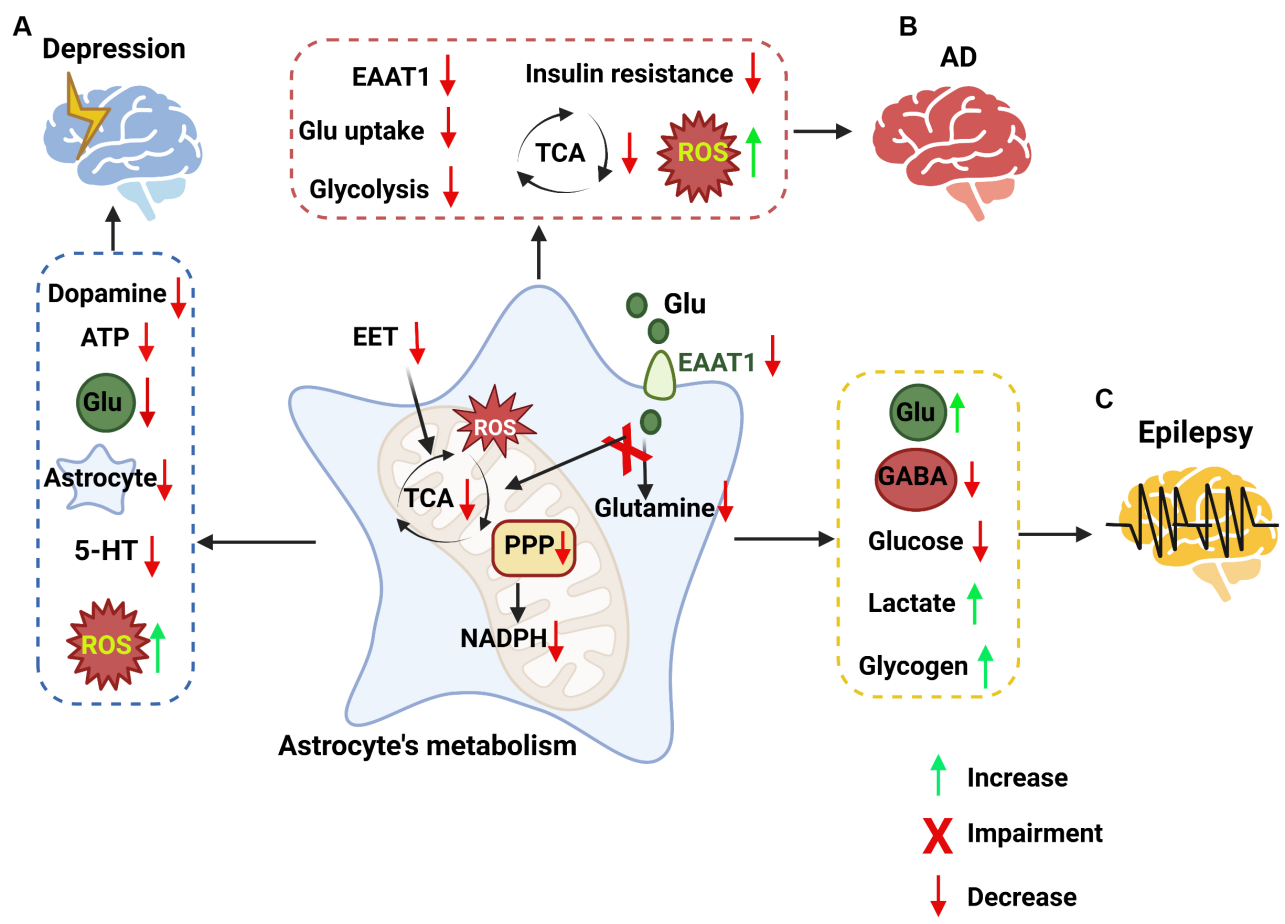
The impacts of astrocyte metabolic pathways on neurological disorders. **(A)** Targeting astrocyte metabolic pathways in depression. In depression, metabolic pathways are impaired in astrocytes, such as epoxyeicosatrienoic acid (EET) signaling, the PPP, the TCA cycle, and an increase in ROS. These changes in astrocytes lead to a decrease in dopamine, ATP, glutamate, astrocytes and 5-HT, while reactive oxygen species (ROS) are increased. **(B)** Targeting astrocyte metabolic pathways in Alzheimer’s disease (AD). Some metabolic signaling is impaired in astrocytes, such as the glutamate uptake pathway, glycolysis pathway, and TCA cycle, while ROS are increased, leading to high inflammation levels. **(C)** Targeting astrocyte metabolic pathways in epilepsy. The pathways involved in epilepsy in astrocytes, such as the metabolism of glutamate, the synthesis of GABA, glycolysis, lactate and glycogen metabolism, were impaired, leading to the accumulation of glutamate, lactate and glycogen and the loss of GABA and glucose.

Astrocytes play a central role in the brain’s metabolic homeostasis, regulating both energy and redox balances (Mulica et al., 2021). In the event of neurological injury, astrocytes can be activated in response to insult. Reactive astrogliosis is a common pathological feature in many neurological disorders and may play a role in neuropathological progression (Zhou B. et al., 2019). Dysfunction of astrocytes and regulatory pathways, including proteins, ion channels, and protein synthesis, may lead to the development of neurological diseases (Pekny et al., 2016; Brandebura et al., 2023). For instance, impairment of astrocyte glutamate uptake and metabolic functions can lead to neuronal excitotoxicity and neurodegeneration (Sun et al., 2021; Satarker et al., 2022). Neurological disorders such as depression, dementia, AD, and epilepsy all show impaired astrocytic metabolism (McDonald et al., 2018; Tournissac et al., 2021). Furthermore, certain neurological imbalances have been associated with reduced glial densities in different brain regions (O'Leary et al., 2021). Changes in glial distributions may cause a shift in the brain’s metabolism. Accumulating evidence suggests that there is a strong correlation between changes in the brain’s metabolism functionality and neurological disorders (Procaccini et al., 2016). Earlier research has revealed a strong connection between astrocyte functionality and neurological diseases. Alterations in astrocytic function, particularly metabolic function, may be a key reason for the worsening of neurological diseases (Figure 4).

### 6.1. Impairment of astrocyte metabolic function in depression

Major depressive disorder (MDD) is a neurological condition caused by chronic exposure to stress. Its characteristics include loss of motivation, impaired social interactions, communication, and pervasive sadness (Luo et al., 2021). Clinical patients with MDD were found to exhibit reduced blood flow and glucose metabolism in the brain (Videbech, 2000). Furthermore, MDD patients have also been found to have impaired TCA cycle functionality, which decreases energy production and may lead to exacerbation of depression-like symptoms (Chen et al., 2019). In MDD, a decrease in the number of astrocytes may lead to an imbalance in neurotransmission, synaptic connectivity, and metabolism (O'Leary and Mechawar, 2021). In postmortem brain tissues of MDD patients, astrocytes were found to have hypertrophic cell bodies and processes in the white matter of the anterior cingular cortex (ACC; Torres-Platas et al., 2011). Experimental models of depression have demonstrated a reduction in the number and density of GFAP-positive astrocytes in the prefrontal cortex (PFC), locus coeruleus, hippocampus, and amygdala, alongside changes in their morphology and functionality (Zhang et al., 2008; Cobb et al., 2016; Rubinow et al., 2016). The reduction in astrocyte density in MDD patients is more prominent than that in neurons (Rajkowska and Miguel-Hidalgo, 2007; Rajkowska and Stockmeier, 2013). In depression, astrocytes undergo morphological alterations characterized by astrocyte atrophy throughout the brain (Zhao et al., 2022). This morphological change may signify impaired functionality of astrocytes in MDD.

Astrocytes are regulators of metabolic energy in the brain. Changes to astrocytic functions in depression primarily revolve around the neuroimmune state, neuronal transmission, and synaptic plasticity. Astrocytes can become reactive when chronically exposed to stress, which can lead to impairment of their functionalities, including intracellular and extracellular ionic regulation, gap junction-based cellular communication, and neurotransmitter metabolism (Guo et al., 2022; Miguel-Hidalgo, 2022). Many studies have indicated that astrocytes play a critical role in regulating various inflammatory signal transductors, such as gp130, transforming growth factor β receptor, interferon-γ receptor, and estrogen receptor α (Colombo and Farina, 2016; Zheng et al., 2021). Astrocytic release of these inflammatory factors can contribute to the development of depressive-like behaviors by causing impaired glutamate uptake (Haroon et al., 2017; Felger, 2018). During depression, inflammation causes the upregulation and release of astrocytic cytokines, which can stimulate a cascade of inflammatory changes, including the activation of proteins such as mitogen-activated protein kinases (MAPK; Ji et al., 2002; Gorina et al., 2011). Activation of the MAPK pathway by inflammation or other stress factors can cause activation of MAPK phosphatase (MKP), which inhibits extracellular signal-regulated kinase (ERK) and elicits depressive-like behavior (Wang and Mao, 2019). Research has revealed that acute inhibition of the ERK pathway has inconsistent results in inducing depressive-like behavior, and chronic pharmacological inhibition of ERK through repeated infusion of the specific MAPK kinase (MEK) inhibitor U0126 into the hippocampus and mPFC has been shown to cause depressive-like behavior (Einat et al., 2003; Duman et al., 2007; Tronson et al., 2008; Qi et al., 2009; Todorovic et al., 2009). Overactivation of ERK has been shown to have antidepressive effects and can alleviate depression (Tronson et al., 2008). Taken together, chronic inhibition of the ERK pathway may be a reason for the development of depression pathology. Depression is a chronic type of disease, and ERK has been shown to have an important link in the development of this pathology. Targeting ERK in astrocytes may elicit antidepressive effects. Astrocytes may also exert antidepressive effects through the release of neuron-protective factors (Li et al., 2021). This shows a fundamental change in astrocytes and their functional changes in neurological disorders.

Astrocytes are connected with neuronal synapses and can influence neuronal excitability through the removal of neurotransmitters such as glutamate, GABA, and purines from the synaptic cleft (Semyanov and Verkhratsky, 2021). In a rat model of depression, the astrocytic potassium channel (K_ir_4.1) drives neuronal bursts in the lateral habenula (LHb), which suggests that it may serve an important function in astrocyte-neuron communication in depression (Cui et al., 2018; Yang et al., 2018). It has been suggested that due to the upregulation of K_ir_4.1, T-type voltage-sensitive Ca^2+^ channels (T-VSCCs) in neurons are activated and initiate NMDAR-dependent neuronal bursts, causing the LHb to trigger depression (Zhao et al., 2022). Although recent research has suggested that the LHb is an important circuit in depression and that constant activation may be a contributing factor to the pathology of depression, it does not take into consideration the altered activation of GABA and Glu neurons in different brain regions.

Another factor that may contribute to the role of astrocytes in depression is the decrease in overall ATP generation and release to neighboring cells. In early research, Cao et al. found that ATP concentrations were drastically lowered in chronic social defeat syndrome (CSDS) mouse models compared with control mice, particularly in the PFC and hippocampus regions of the brain (Cao et al., 2013; Wang et al., 2021). More recently, Xiong and his team found that impaired epoxyeicosatrienoic acid (EET) signaling can impede ATP release from astrocytes in the mPFC, inducing depressive-like behavior (Xiong et al., 2019). The dynamics of ATP release from astrocytes are crucial in preventing depressive-like behavior. ATP released from astrocytes can be used to modulate a plethora of functions, including various brain activities. ATP is released by astrocytes through Ca^2+^ flux, which has been found to regulate axon excitability (Lezmy et al., 2021). ATP can also influence the release of glutamate from astrocytes, thereby causing changes in neuronal modulation (Jeremic et al., 2001). Research has also found that impairment of the glutamate metabolic pathway can also lead to increased depression-like behavior (Lee et al., 2013). Impairment of the glutamate metabolic pathway can negatively affect dopaminergic neurons by insufficiently inhibiting kynurenine, causing decreased dopamine release (Kulagina et al., 2001). Furthermore, the decrease in neurotransmitters, including glutamate and dopamine, may result in decreased pyramidal neuron firing (Vitrac et al., 2014).

In addition to dopamine, serotonin is also reduced primarily due to the decrease in cholesterol levels in the body due to decreased appetite and body weight (Sun et al., 2015). This is important because cholesterol and blood lipids can decompose to form a substrate triose phosphate that can then be converted into pyruvate and enter the TCA cycle (Gu et al., 2021). In recent research, impairment of mitochondrial functionality was found in humans with MDD, such as lowered respiration and ATP-related oxygen consumption (Kuffner et al., 2020). In MDD mice, high levels of reactive oxygen species (ROS) were found, which may be due to NADPH deficiency due to impaired PPP. Notably, a decrease in glutathione in the PPP may also result in the accumulation of ROS that trigger oxidative stress, causing inflammation and possibly contributing to the worsening of depression (Ozaslan et al., 2019). This demonstrates the importance of astrocyte metabolism in neuronal functionality and depression.

### 6.2. Astrocyte metabolism and AD

Astrocytes’ metabolic pathways provide energy to neurons for various functions through neuron modulation, such as memory, motor, and cognitive functions (Padmashri et al., 2015; Santello et al., 2019; Lines et al., 2020). AD is a neurogenerative disorder that is characterized by progressive cognitive decline, loss of memory, and dementia. There are many theorized causes of AD, one of the main causes being metabolic dysfunction (Cai et al., 2012). Significant metabolic coupling is present between astrocytes and neurons, especially during synaptic activity (Magistretti, 2006). In the onset stages of the AD mouse model, there was progressive astrocytic atrophy with decreased GFAP staining in the cortex and hippocampus of the brain (Yeh et al., 2011; Beauquis et al., 2013). However, in postmortem tissue of AD patients, it was found that there was progressive astrocytic hypertrophy and upregulation of GFAP (Simpson et al., 2010). The change in astrocyte morphology and a switch from an atrophic phenotype to a hypertrophic phenotype may be associated with the accumulation of Aβ. It is well accepted that in late stages of AD, the functionality of astrogliosis mostly revolves around Aβ clearance (Guenette, 2003; Nicoll and Weller, 2003). Astrocytes also play an integral role in regulating vasoconstriction and vasodilatation (Iadecola and Nedergaard, 2007). Through these two functions, it may be possible for reactive astrocytes to contribute to damage to the neurovascular unit at the onset of AD.

One of the main factors contributing to AD is the genetic risk of apolipoprotein E (APOE), mainly expressed in astrocytes, which contributes to the accumulation of β-amyloid in the brain (Verghese et al., 2013; Arranz and De Strooper, 2019). Additionally, genes such as clusterin and fermitin family member 2, also expressed in astrocytes, are also closely related to AD (Preman et al., 2021). This emphasizes the importance of astrocytes in AD and the importance of considering their role in the disease. Earlier research showed that inhibition of astrogliosis exacerbated Aβ accumulation and pathology in AD mice (Kraft et al., 2013). Reactive astrocytes in regions with plaque buildup showed impaired Ca^2+^ dynamics (Kuchibhotla et al., 2009; Agulhon et al., 2012). Astrocyte Ca^2+^ hyperactivity can promote the release of detrimental factors, alter neuronal-glial communication, and impair synaptic transmission (Frost and Li, 2017; Verkhratsky et al., 2017a). Recent hypotheses suggest that astrocytes could be involved in Aβ production, as they upregulate β-secretase 1 and amyloid precursor protein (APP) in AD brains (Frost and Li, 2017). However, there are currently no definitive data pointing to astrocytes as a major source of β-amyloid. Instead, astrocytes may mainly participate in β-amyloid clearance through various mechanisms, such as producing β-amyloid-degrading proteases, extracellular APOE, ApoJ/Clusterin, α1-antichymotrypsin (ACT) and α2-macroglobulin (α2-M; Ries and Sastre, 2016; Preman et al., 2021). Mutation and dysfunction of astrocytes in the expression or regulation of these proteins during AD may be a reason for the altered clearance processes of Aβ.

In AD, astrocytes may shift the excitation-inhibition balance through the secretion of GABA (Jo et al., 2014). Normally, astrocytes in the brain do not contribute to GABA production; however, in AD, GABA is synthesized through the astrocytic putrescine-monoamine oxidase B pathway (MAO-B; Jo et al., 2014). Hypothetically, astrocytic GABA release may be a defensive mechanism to protect neurons from further harm that may arise from excitotoxicity caused by AD (Ghatak et al., 2019). Although GABA synthesis may initially support neuron survival, the increase in MAO-B expression for GABA synthesis may result in elevated production of hydrogen peroxide, which may worsen the condition (Chun et al., 2020).

In AD patients, the expression of EAAT1 and EAAT2 in brain astrocytes was found to be reduced, which can lead to impaired neuronal functionality (Liang et al., 2002). Neurodegeneration was also found to involve this mechanism, in which astrocytes with impaired glutamate uptake possessed lower EAAT2 and GLAST expression (Hefendehl et al., 2016). Impairment of the glutamate metabolic system may be one of the reasons for continuous memory loss and confusion in AD patients. Another metabolic change is the decrease in the brain’s glucose uptake and glycolysis, which can be viewed as the early onset of AD (Ding et al., 2013; Tomi et al., 2013). It was found that metabolism-related genes, such as those responsible for the regulation of the glycolytic pathway and TCA cycle, were significantly downregulated in both an AD mouse model and AD patients (Chen et al., 2012). The activity of glucose 6-phosphate dehydrogenase was found to be significantly decreased, while lactate dehydrogenase increased in the frontal and temporal cortexes in AD patients (Yun and Hoyer, 2000). Moreover, patients who have suffered from frontotemporal dementia exhibited glucose hypometabolism in the cortical regions of the brain (Garrett and Niccoli, 2022).

The hypometabolism of glucose may also be attributed to insulin resistance (Kang et al., 2017). Insulin was found to regulate glucose uptake and metabolism in astrocytes, and insulin resistance may be a contributing factor for AD (Fernandez et al., 2017). Overproduction of insulin can affect astrocytes and Aβ accumulation by saturating insulin-degrading enzyme (IDE), which was also found to degrade Aβ (Kang et al., 2017). Aβ accumulation is one of the hallmarks of AD and has been found to alter metabolic pathways in the brain (Fu and Jhamandas, 2014). It was found that Aβ aggregates and is internalized into astrocytes through scavenger receptors located on the plasma membrane, which alters glucose metabolism. The accumulation of Aβ in astrocytes is responsible for increased ROS production and decreased glutathione levels, leading to oxidative stress and neuronal vulnerability (Allaman et al., 2010). Aβ accumulation has been found to be a key player in activating microglia and downregulating CX3C motif chemokine receptor 1 (CX3CR1; Grubman et al., 2019; Muzio et al., 2021). This activation may trigger synaptic neurotoxicity and neurodegeneration (Hansen et al., 2018; Dejanovic et al., 2022). Notably, inhibition of this pathway has been found to alleviate synapse loss and neurodegeneration in murine models of AD (Wu et al., 2019; Dejanovic et al., 2022). Recent research has found a bidirectional interaction between the nervous system and immune system, signifying that systemic inflammation could cause selective neuronal activation (Brea and Veiga-Fernandes, 2022). A pathological aspect of AD is that a breakdown of the BBB occurs, causing the infiltration of toxicants and immune cells into the brain (Sweeney et al., 2018). The degradation of the BBB in AD pathology is one of the causes of neuroinflammation and results in the activation of downstream cascades associated with neural injury and neurodegeneration (Sweeney et al., 2018). Postmortem analysis of AD patient brains revealed an accumulation of metal ions such as iron (Fe) and zinc (Zn) due to dysregulation (Lovell et al., 1998). These metal ions are found to colocalize with Aβ aggregates, suggesting that Aβ may cause the accumulation of Fe and Zn, which in turn induces ferroptosis and AMPAR-mediated neurotoxicity (Weiss et al., 1993; Cheng et al., 2021). In more recent studies, it has been suggested that elevated epoxide hydroxylases in the brain could contribute to neuroinflammation observed in AD (Ghosh et al., 2020). These epoxide hydroxylases can bind to and inhibit anti-inflammatory arachidonic acid derivatives, thereby promoting inflammation (Ghosh et al., 2020; Pathak and Sriram, 2023a). It is interesting to note that Aβ accumulation can alter glucose metabolism as well as hydrogen peroxide production and glutathione release in cultured astrocytes, showing that ROS are produced through astrocytic metabolic dysfunction. The toxic effect of Aβ on astrocytes is mainly expressed through mitochondrial depolarization and loss of Ca^2+^ homeostasis (Abramov et al., 2004). Astrocyte mitochondrial dysfunction can influence the homeostatic transport of Na^+^/K^+^-ATPase, thereby driving the accumulation of neurotransmitters such as glutamate and GABA (Genda et al., 2011; Jackson et al., 2014). ATP deficiency may affect glutamate clearance and thereby promote excitotoxicity (Preman et al., 2021). Taken together, the alteration to astrocytic glucose metabolism due to Aβ accumulation and its effects on neighboring neurons points to metabolic alterations as being a key culprit in the development of AD.

### 6.3. Astrocyte metabolism and epilepsy

Astrocytic regulation of metabolic function is paramount in the role it plays in epilepsy. Epilepsy is caused by the imbalance of excitatory and inhibitory neurons in the brain, which may be a cause of metabolic dysfunction (Reddy and Saini, 2021; Qi et al., 2022). Astrocytes can participate in neurotransmission by regulating ion concentrations and neurotransmitters (Tritsch and Bergles, 2007). In epilepsy, astrocytes adopt a reactive morphology (Heinemann et al., 2000; Binder and Steinhauser, 2021), become uncoupled (Bedner et al., 2015), and lose domain organization (Oberheim et al., 2008). These changes can have a variety of influences on functionality. These morphological changes may lead to dysfunctions in glutamate clearance (Coulter and Eid, 2012). Changes to astrocytic functionalities in epileptic conditions may exacerbate epileptic symptoms. During epilepsy, the increase in K^+^ flux may result from ion channel dysregulation. Research has found that in epilepsy, downregulation of K_ir_4.1 reduces astrocytes’ ability to take up glutamate and K^+^ from the extracellular environment, leading to increased seizures (Djukic et al., 2007; Chever et al., 2010; Haj-Yasein et al., 2011). Structural analysis of astrocytes showed spatial overlap of the K^+^ channel K_ir_4.1 and aquaporin-4 (AQP4; Nielsen et al., 1997; Higashi et al., 2001). This research suggests that K^+^ uptake through K_ir_ channels may depend on osmotic flux. This can indirectly affect the uptake and clearance of glutamate by astrocytes. The accumulation of glutamate in the brain due to the lack of uptake and clearance by astrocytes may be a key factor in epileptogenesis.

In temporal lobe epilepsy (TLE) patients, increased interictal glutamate levels and increased seizure-induced glutamate transients were found in the hippocampus (Cavus et al., 2005). This may be due to the impaired uptake of glutamate in the brain. A decrease in glutamine synthase was also discovered in epilepsy patients, suggesting that even after glutamate enters astrocytes, the clearance of glutamate may still be limited (Eid et al., 2019). Glutamate uptake into astrocytes can trigger astrocytic glycolysis (Bittner et al., 2011). During excessive synaptic activity, a decrease in glucose and a rise in lactate were found, signifying that lactate becomes the primary energy source for neurons during energy-intensive activities (Boison and Steinhauser, 2018).

In clinical settings, patients with TLE were found to also have increased glucose uptake and metabolism during seizures, whereas it is severely reduced during the interictal period (Engel Jr. et al., 1983). In addition to lactate being a viable fuel source for driving hippocampal epilepsy, glycogen stored in astrocytes can be transported into neurons through the lactate shuttle and converted into lactate for fuel (Boison and Steinhauser, 2018). It was found that in a methionine sulfoximine (MSO)-induced epilepsy mouse model, glycogen was rapidly metabolized during seizures but returned to normal during the interictal phase (Bernard-Helary et al., 2000). In MSO-induced epilepsy, the activity of the glutamate reuptake pathway was found to decrease, resulting in increased neuronal excitability (Eid et al., 2008). Decreased activity of glutamate reuptake can also lead to loss of inhibition by GABAergic neurons due to impaired GABA synthesis and release (Liang et al., 2006). Astrocytes are found to form coupled networks of cells for various functions (Wallraff et al., 2006). These networks allow for the exchange of ions, second messengers, metabolites, and amino acids from astrocytes to neurons. Glucose trafficking through coupled astrocytes is necessary for hyperactivity, while extracellular glucose deprivation causes loss of synaptic hyperactivity that can be rescued when astrocytes are filled with glucose or lactate, showing the importance of metabolism in epilepsy formation. Furthermore, astrocytes were found to be involved in regulating neuronal synchronization and the spread of ictal activity through Ca^2+^ channels in the gap junction (Gomez-Gonzalo et al., 2010). In contrast to their involvement in Ca^2+^ channel modulation, astrocytes are also thought to possess antiepileptic functions because reduced astrocytic coupling was found to cause extracellular K^+^ and glutamate build-up, resulting in depolarization and seizure generation (Pannasch et al., 2011; Boison and Steinhauser, 2018). Taken together, astrocytes possess both pro-epilepsy and anti-epilepsy properties. It has been shown to be a regulator of glutamate homeostasis, while the glucose metabolic pathway is crucial for the development of epilepsy.

In contrast to dysfunction of astrocyte metabolism in epilepsy, inflammation has also been a contributing factor for the development of epileptogenesis (Hayatdavoudi et al., 2022). In response to neuronal injury caused by excitotoxicity, astrocytes can generate and release cytokines such as IL-1β, IL-6, tumor necrosis factor (TNF)-α, transforming growth factor (TGF)-β, monocyte chemoattractant protein-1 (MCP-1), and chemokine C-motif ligand 2 (CCL2; Giovannoni and Quintana, 2020; Kwon and Koh, 2020). These signals were found to be highly expressed in both experimental and human epileptogenic brain tissues, indicating that these inflammatory signals may be tied to epilepsy pathology (Aronica and Crino, 2011; Giovannoni and Quintana, 2020). TNF-α secreted by microglia can induce astrocyte reactivity (Chen et al., 2021). Additionally, TNF-α has been shown to regulate neuronal activity and induce epilepsy by increasing glutamate neurotransmitter release (Shim et al., 2018). In earlier research, IL-1β release and activation through interleukin-converting enzyme (ICE) and caspase-1 may contribute to acute seizures and drug-resistant chronic epilepsy in mice (Maroso et al., 2011). Pharmacological inhibition of IL-1B synthesis using VX-765 has been found to reduce epileptic activity (Maroso et al., 2011). Astrocyte and microglial release of these cytokines may be a main contributing factor to epileptogenesis. Targeting inflammatory cytokines may be another effective option in epilepsy treatment, particularly in patients who have developed refractory epilepsy.

## 7. Conclusion and outlook

Astrocytes are an integral element of neurobiology and have been rapidly revealing themselves as more than a mere supportive player in the complexity of the brain’s neural circuit. Their intricate roles extend far beyond the basics of metabolic regulation and hold the potential to unlock novel insights into the complex etiology and progression of various neurological disorders, including depression, Alzheimer’s disease, and epilepsy. This emerging perspective compels a comprehensive reconsideration of our understanding of astrocytes. In fact, their central role in neurological health and disease suggests that in-depth exploration of their function is not only important but necessary to develop effective treatments for these conditions. Projecting into the future of neuroscience, the trajectory of astrocytic research appears clear and promising. Deeper exploration into the nuanced interaction between astrocytes and neurons, their symbiotic metabolic relationship, and the potential to restore their function in pathological states can open new avenues for the treatment and prevention of diverse neurological disorders. In conditions such as depression and AD, emerging research suggests that dysfunctional energy metabolism in terms of glucose utilization in astrocytes may be a pivotal factor that warrants further research. Another aspect worth researching is the dual role of astrocytes in neurological disorders. Whether astrocyte activation is beneficial in AD is still unclear. Targeting astrocytic metabolism holds immense potential for the development of therapeutic interventions targeting metabolic abnormalities. Similarly, in epilepsy, the role of astrocytes in regulating ion balance by modulating ion channels and neuronal communication appears to be compromised, implying that therapeutic strategies aiming to rectify these disruptions could be beneficial in controlling epileptic seizures. Interestingly, astrocytes seem to be a double-edged sword in regard to their behavior in epilepsy, acting both as protective agents and instigators. Thus, future research needs to focus on comprehending this dual role and how we can potentially manipulate it for use in therapeutic treatments. As we continue to make technological strides in our exploration of astrocytes, the potential to elucidate their complex roles increases. Understanding their unique vulnerabilities and harnessing their innate potential could unveil novel treatments and significantly improve the prognosis for individuals afflicted with neurological disorders. To summarize, the future trajectory of astrocyte research is replete with promise and potential that warrants further research.

## Author contributions

Y-mZ and Y-bQ conceived the idea, wrote the original manuscript, drew the figures and revised the manuscript. Y-nG, W-gC, and TZ edited the initial draft and revised the manuscript. JL and YZ provided financial support and revised the manuscript. All authors contributed to the article and approved the submitted version.

## Funding

This review was supported by grants from the National Natural Science Foundation of China (81971265).

## Conflict of interest

The authors declare that the research was conducted in the absence of any commercial or financial relationships that could be construed as a potential conflict of interest.

## Publisher’s note

All claims expressed in this article are solely those of the authors and do not necessarily represent those of their affiliated organizations, or those of the publisher, the editors and the reviewers. Any product that may be evaluated in this article, or claim that may be made by its manufacturer, is not guaranteed or endorsed by the publisher.
