# Supplementary material for: Astrocyte metabolism and signaling pathways in the CNS
Source: Front Neurosci. 2023 Sep 4;17:1217451. doi: 10.3389/fnins.2023.1217451 (PMC10507181; doi:10.3389/fnins.2023.1217451)
Supplement: Supplementary file 1 [file Data_Sheet_1.PDF]

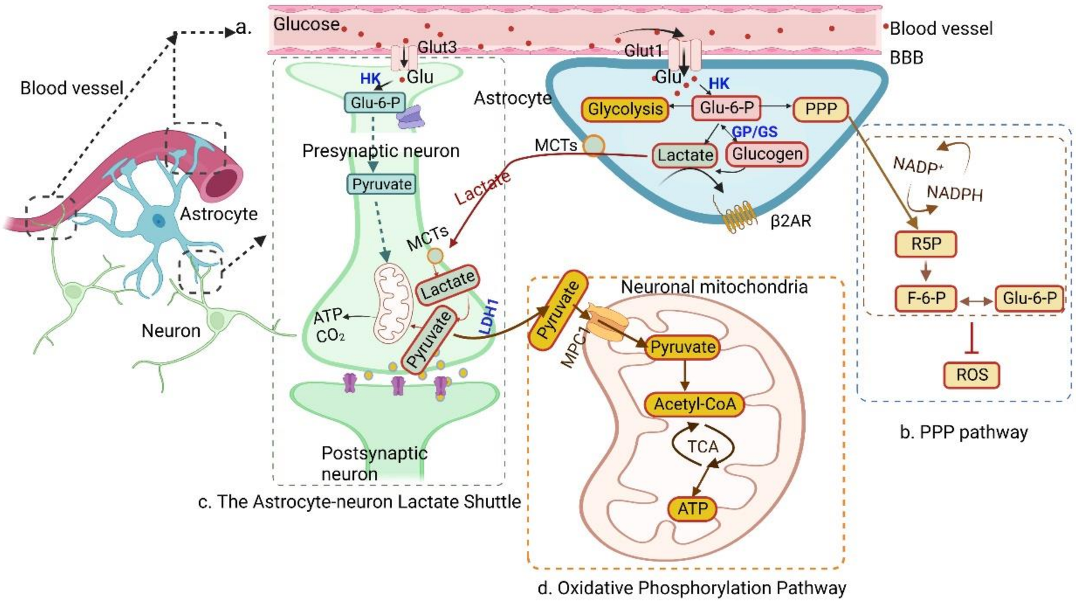

Figure 1. The glucose metabolism in astrocytes. a. Glucose is transported from the blood brain barrier (BBB) to astrocyte and neurons through glucose transporters (GLUT), and phosphorylated to glucose-6-phosphate (G6P). G6P enter different pathways such as: glycolysis, the astrocyte-neuron lactate shuttle, the pentose-phosphate pathway (PPP), and oxidative phosphorylation pathway. **b**. In the PPP pathway, G6P catalyzes to ribulose-5-phosphate (R5P) converting nicotinamide adenine dinucleotide phosphate (NADP)+ to NADPH at the same. Concurrently, R5P can also be converted to glyceraldehyde-3-phoshpate and fructose-6-phosphate (F6P), the latter can isomerize back to G6P. **c.** The astrocyte-neuron lactate shuttles provide energy for neuron activity. Lactate is transferred from astrocytes into neurons through monocarboxylic acid transporters (MCTs) and is converted to pyruvate to generate ATP in mitochondria. **d.** The oxidative phosphorylation pathway in astrocyte converts G6P to pyruvate, which undergoes oxidative decarboxylation to form acetyl-CoA, and then enters the tricarboxylic acid (TCA) cycle to generate ATP in mitochondrial. MPC1, mitochondrial pyruvate carrier 1. HK: hexokinase; GP: glycogen phosphorylase; LDH1: lactate dehydrogenase 1.

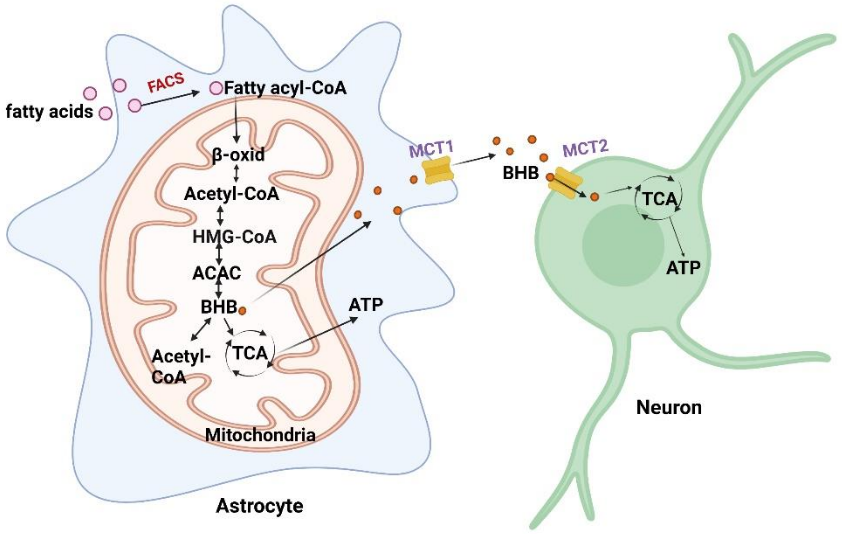

Figure 2. The fatty acid metabolism in astrocytes. When energy is scarce, fatty acid is converted to fatty acyl-CoA and undergoes β-oxidation (β-oxid) to produce β-hydroxybutyrate (BHB). BHB converts back to acetyl-CoA via β-ketoacyl-CoA transferase or enters the TCA cycle to generate ATP. It then enters the TCA cycle to generate ATP. FACS, fatty acyl-CoA synthetase; HMG-CoA, 3-hydroxy-3-methylglutaryl coenzyme A; ACAC, acetoacetate.

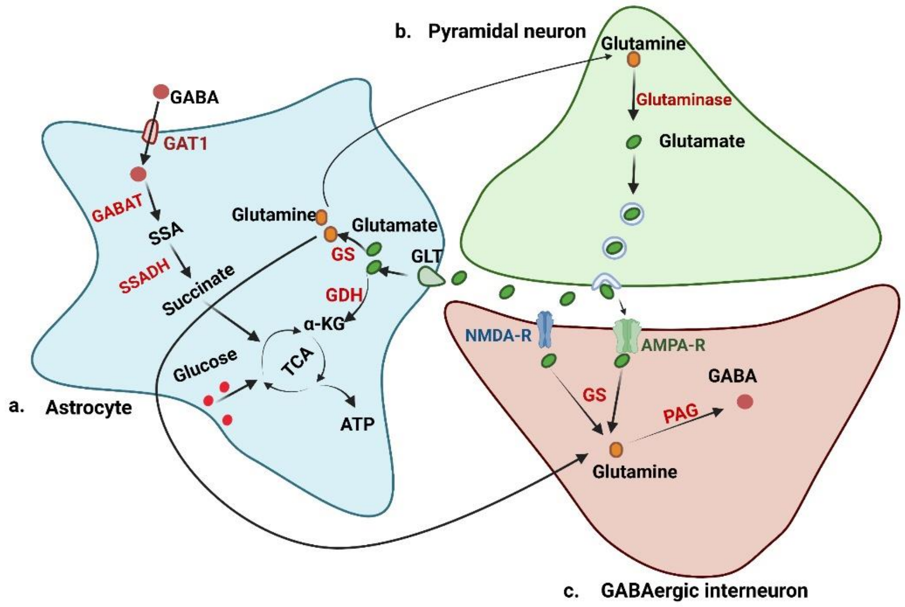

Figure 3. The glutamate and GABA metabolism. a. Astrocytes regulate glutamate in the brain through glutamate transporter (GLT). After being transported in astrocyte, glutamate undergoes glutamine synthase pathway to produce glutamine or TCA cycle to generate ATP. **b.** The glutamate synthesis pathway in neurons. Glutamine in neurons is transported from astrocytes and is deaminized to generate glutamate by glutaminase. **c.** The GABA synthesis in neurons. The glutamine in neurons for GABA synthesis is transported from astrocytes or synthesized from glutamate which is released from the excitatory synapse. Glutamine is then converted to GABA through phosphate-activated glutaminase (PAG). GS, glutamine synthetase; GDH, glutamate dehydrogenase; GABAT, GABA transaminase; GAT1, GABA transporter 1; SSA, succinyl semialdehyde; SSADH, semialdehyde dehydrogenase.

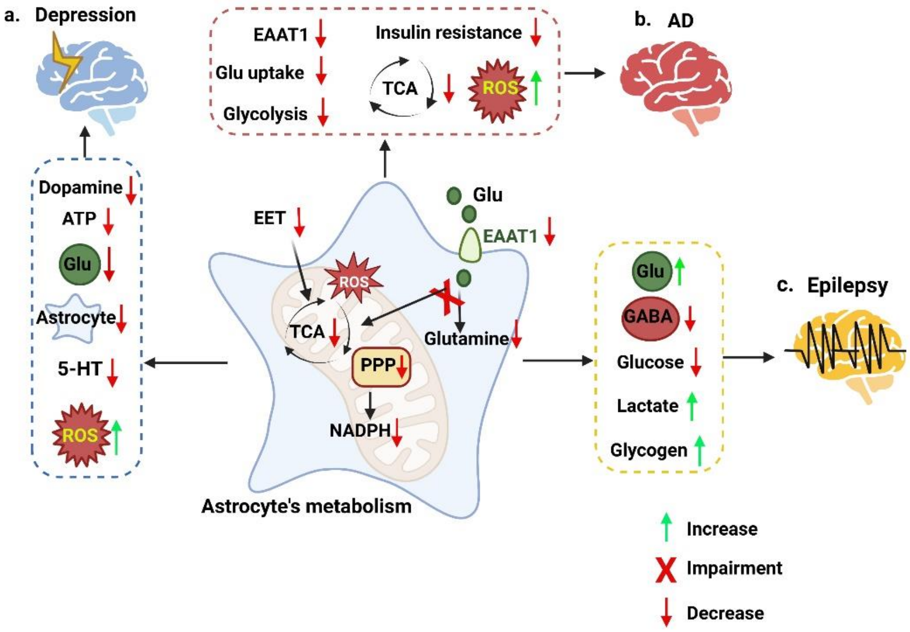

Figure 4. The impacts of astrocyte's metabolic pathways on neurological disorders. a. Targeting astrocyte's metabolic pathways in depression. In depression, the metabolic pathways are impaired in astrocytes, such as the epoxyeicosatrienoic acid (EET) signaling, PPP, the TCA cycle, and increase in ROS, those changes in astrocytes leads to the decrease of dopamine, ATP, glutamate, astrocyte and 5-HT, while the reactive oxygen species (ROS) is increased. **B.** Targeting astrocyte's metabolic pathways in Alzheimer's disease (AD). Some metabolic signaling is impaired in astrocytes, such as, the glutamate uptake pathway, glycolysis pathway, TCA cycle, while ROS is increased leading to the high inflammation level. **C.** Targeting astrocyte's metabolic pathways in epilepsy. The pathways involved in epilepsy in astrocyte such as: the metabolism of glutamate, the synthesis of GABA, glycolysis, the lactate and glycogen metabolism were impaired, leading to the accumulation of glutamate, lactate and glycogen, and the loss of GABA and glucose.
